# Is Primary Bone Marrow Edema of the Knee Associated with Thyroid Disorders? A Retrospective Clinical Study

**DOI:** 10.3390/jcm11195973

**Published:** 2022-10-10

**Authors:** Luca De Berardinis, Fjorela Qordja, Luca Farinelli, Andrea Faragalli, Rosaria Gesuita, Antonio Pompilio Gigante

**Affiliations:** 1 Clinical Orthopaedics, Department of Clinical and Molecular Science, School of Medicine, Università Politecnica delle Marche, Via Tronto, 10/a, 60126 Ancona, Italy; 2 Centre of Epidemiology, Biostatistics and Medical Information Technology, Università Politecnica delle Marche, Via Tronto, 10/a, 60126 Ancona, Italy

**Keywords:** bone marrow edema, knee, thyroid disorders, anxiety-depressive disorders, COVID-19, NSAIDs

## Abstract

Primary bone marrow edema (BME) of the knee is still an elusive condition. This retrospective study was undertaken to gain insight into its characteristic features. The records of 48 patients with primary BME of the knee diagnosed by magnetic resonance imaging were reviewed. Demographic data, medical history, current medications, pain type, smoking and drinking habits, allergies, occupation, sports practiced, environmental factors, and life events predating symptom onset were examined. Data analysis demonstrated that 56.3% of patients had experienced a stressful event before BME pain onset and that 50% suffered from thyroid disorders. Standard conservative treatment resulted in pain resolution irrespective of the use of anti-inflammatories. However, most patients reported new persistent symptoms: dysesthesia/hypoesthesia on palpation in the skin area overlying the previous edema and a reduced ipsilateral patellar reflex. To our knowledge, this is the first study characterizing a substantial cohort of patients with BME. We found that middle-aged, sedentary, and slightly overweight women smokers are the typical patients with primary BME of the knee. The appearance and persistence of cutaneous dysesthesia/hypoesthesia at the site of the earlier lesion and ipsilateral patellar hyporeflexia implicate an autonomous nervous system dysfunction in BME pathogenesis and warrant further investigation.

## 1. Introduction

The term bone marrow edema (BME) was introduced by Wilson and co-workers [1] to describe a radiological finding often detected in patients suffering from joint pain, namely decreased bone marrow signal intensity on T1-weighted images and increased signal intensity on T2-weighted images on magnetic resonance imaging (MRI). The resulting BME syndrome [1] has since been denominated algodystrophy, reflex sympathetic dystrophy, transient osteoporosis, regional migratory osteoporosis, transient bone marrow edema, bone marrow lesion, and bone marrow edema-like lesion [2,3,4,5]. Nonetheless, the finding is not specific, since it is also described in a wide range of conditions having diverse histopathological features and etiologies including vascular, traumatic, and inflammatory disorders [6,7,8,9].

BME may be primary or secondary. The etiology and pathogenesis of primary BME are unknown. Intramedullary augmentation of intraosseous pressure, with nociceptive fibers triggering the characteristic pain during walking and at night [5,10], and vitamin D deficiency [10,11,12,13] are among the hypotheses advanced to explain it. Secondary BME is found in patients suffering from conditions such as osteonecrosis, osteochondritis dissecans, complex regional pain syndrome, osteoarthritis, mechanical strain such as bone contusion (bone bruise), micro-fracture and stress fracture, and primary or metastatic tumors [14].

Treatment is closely related to the etiology and extent of the bone necrosis. Osteonecrotic lesions exceeding 40–50% of the femoral or tibial condyle area or larger than 5 cm^2^ usually lead to bone collapse and require arthroplasty [15]; medium-sized lesions (3.5–5 cm^2^) may regress, whereas smaller lesions (<3.5 cm^2^) are usually managed non-operatively [16]. Conservative treatment includes analgesics, non-steroidal anti-inflammatory drugs (NSAIDs), weight-bearing restrictions, physiotherapy, pulsed electromagnetic fields (PEMFs) [17], prostacyclin, and bisphosphonates [17]. Surgical management is reserved for the late disease stages and involves simple perforation, fragment stabilization, scraping and perforation, and eventually osteochondral/chondrocyte transplant [9].

This retrospective study was undertaken to analyze the demographic and clinical characteristics of a cohort of patients with primary BME of the knee, identify possible risk factors, and evaluate treatment outcomes.

## 2. Materials and Methods

**Figure 1:**
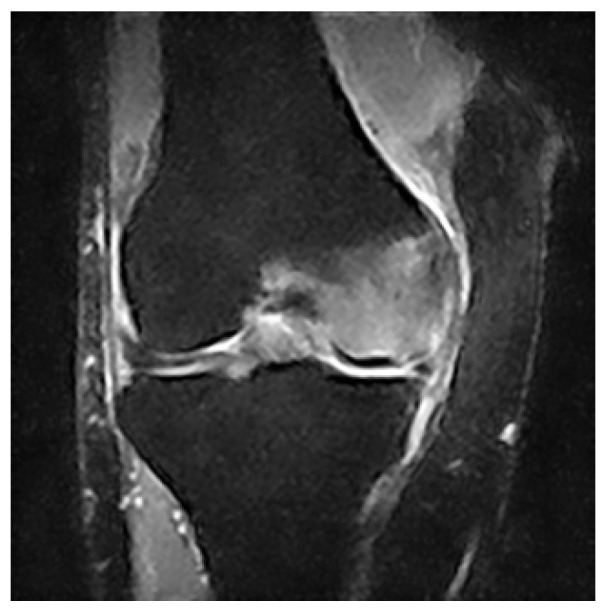
Coronal MRI image showing a typical BME pattern of our patients.

The archives of our institution were mined for the records of patients who had been diagnosed with primary BME of the knee from 1 January 2015 to 31 August 2021. The diagnosis was based on the sudden onset of acute non-traumatic knee pain and on abnormal bone marrow signal strength on MRI, specifically decreased bone marrow signal intensity on T1-weighted images and increased signal intensity on T2-weighted images, as presented in Figure 1.

The scans had been obtained at different institutions in magnets with a field strength of 3 Tesla or higher using the same imaging protocols and were reviewed simultaneously by two musculoskeletal radiologists and two orthopedic surgeons with 10 years of experience.

All patients provided their informed consent to the use of their medical records and personal data at the time of admission. Ethical committee approval was not required for the production of this retrospective article. The study was performed in accordance with relevant guidelines and regulations and the Declaration of Helsinki, as revised in 2013.

The inclusion and exclusion criteria are summarized in Table 1.

**Figure 2:**
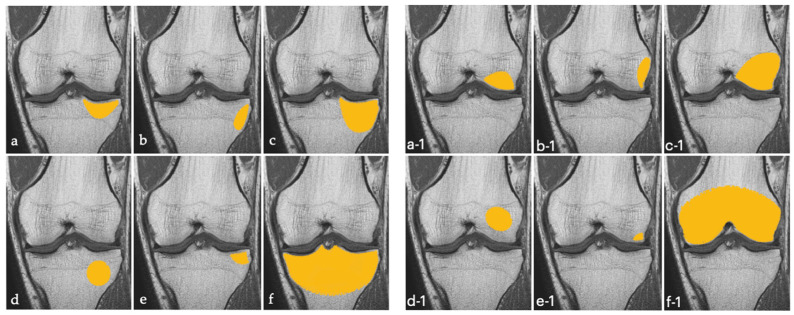
Schematic illustration of tibial (on the left (**a**–**f**)) and femoral (on the right (**a-1**–**f-1**)) primary bone marrow edema of the knee, in according to Compagnoni’s classification [18].

We defined BME of the knee according to the topographic classification proposed by Compagnoni et al., well-illustrated in Figure 2 below [18].

Medical histories with emphasis on known and suspected risk factors for abnormal bone metabolism, type of knee pain, current medications, allergies, age, gender, body mass index (BMI), smoking and drinking habits, occupation, sports practiced, geographic area of residence, life events closely preceding symptom onset, and clinical outcomes were examined.

All patients had been managed by the same treatment protocol: PEMFs applied to the lateral side of the knee (8 h at night for 30 days with voltage pulses of 1.3 ms, 75 Hz) [19,20], oral anti-inflammatories (ibuprofen 600 mg, twice daily for 15 days), oral vitamin D3, vitamin K2, and calcium supplements (5 g sachets, once daily for 20 days), intramuscular bisphosphonates (clodronic acid 200 mg/4 mL, 1 daily injection for 10 days, then 1 injection on alternate days for 20 days), and oral analgesics (paracetamol 1000 mg, as needed) [21].

### Statistical Analysis

A descriptive analysis of patient data, knee pain, and radiological findings was performed. Median and interquartile range (IQR) and absolute and percentage frequencies were used to summarize quantitative and qualitative variables, respectively. The monthly distribution of BME onset was recorded. A non-parametric analysis of longitudinal data in factorial experiments was applied to evaluate pain before and after therapy in patients treated and not treated with NSAIDs using a numerical rating scale (NRS). The interaction between groups and BME duration was also assessed.

To test the possible association between thyroid disorders (exposure) and BME (outcome), odds ratios were calculated to compare the 48 patients with primary BME of the knee with (i) all the patients with a variety of orthopedic conditions who attended our clinic in the study period and (ii) with those whose orthopedic conditions did not involve the knee, to exclude any bias related to the knee site.

## 3. Results

**Figure 3:**
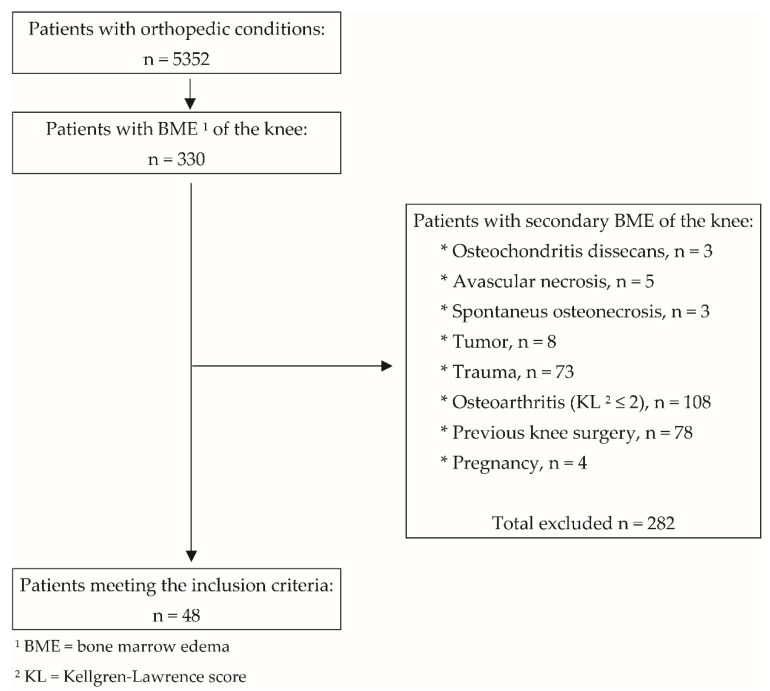
Patient selection protocol according to the inclusion and exclusion criteria.

Of 5352 patients who were referred to our clinic from 1 January 2015 to 31 August 2021, 330 were diagnosed with BME of the knee and 48 had primary BME of the knee (Figure 3). All had complete bone marrow lesions on MRI, i.e., lesions involving the articular and medial or lateral cortex of the condyle that exceeded 50% of the medial or articular surface or crossed the physis [18].

### 3.1. Age, Habits, and Clinical History

Of the 48 patients, 56.2% were women (median age, 63 years; IQR, 59.5–68.5). Median BMI was 26.8 kg/m^2^ (IQR, 24.1–29.4); the median number of pregnancies was two (IQR, 2–2) and the median age at menopause was 45 years (IQR, 44–49). Altogether, 43.8% of patients suffered from allergies to drugs, foods, or metals, 68.8% were smokers, and 75% drank alcohol with their meals. In addition, 68.8% of patients were teachers, employees, or shopkeepers who did not engage in heavy manual work and 81.3% had never practiced any sport.

Half of the patients suffered from thyroid disorders, which in 62.5% of cases were managed with thyroid replacement medication. In addition, 12.5% and 50.0% of patients had type 2 diabetes mellitus and hypertension, respectively; 68.8% of patients used proton pump inhibitors. Most patients (27; 56.2%) came from the north of Italy, followed by southern and central Italy (15; 31.3% and 6; 12.5%, respectively).

### 3.2. Knee Pain

**Figure 4:**
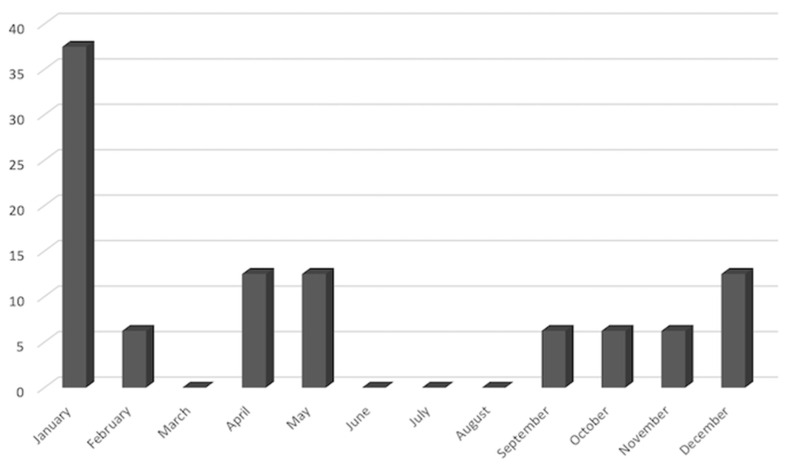
Monthly distribution of knee pain onset.

All patients described their knee pain as sudden. As shown in Figure 4, BME pain arose more frequently from December to February, the coldest months of the year. In nearly all patients (45; 93.8%) the pain was continuous throughout the day and night; 20% and 13.3% of patients described it as burning and throbbing, respectively.

Pain intensity was rated on a 1–10 NRS scale. The median value before treatment was 8.5 (IQR, 8–10). Pain was almost continuous (42 patients; 87.5%) during active movement as well as during passive flexion and extension of the knee; in 87.5% of cases it was not localized but it involved the whole knee. The pain was largely disabling, as only 37.5% of the knees were capable of weight-bearing.

Median pain duration since the inception of treatment was 4 months (IQR, 3–4). The pain resolved in 42 patients (87.5%) and fell to NRS 3 (three patients) or 4 (three patients).

### 3.3. Life Events Preceding the Onset of Knee Pain

More than half of the patients (56.3%) had suffered from very stressful events between 7 and 10 days before pain onset. The events included bereavement in the family (66.7%), work-related problems (20.8%), and anxiety and fear due to the COVID-19 pandemic (12.5%).

### 3.4. Radiological Findings

There was no side predominance in the painful knee. All patients had complete (type C) lesions [18] that involved the medial tibial plateau (43.8%), the lateral tibial plateau (6.2%), the medial femoral condyle (31.2%), or the lateral femoral condyle (18.8%).

### 3.5. Treatment Outcomes

The clinical outcomes are summarized in Table 2. Complete regression of the edema was documented in the MRI scans from all patients. Nonetheless, 45 (93.8%) patients described the appearance and persistence of three new symptoms: dysesthesia and hypoesthesia on palpation in the skin area overlying the earlier edema and a reduced ipsilateral patellar reflex.

After treatment, the pain score fell significantly (*p* < 0.001) in all patients, irrespective of NSAID administration (Table 3).

To test the possible association between thyroid disorders (exposure) and BME (outcome), odds ratios were calculated to compare the 48 patients with primary BME of the knee with (i) all the patients with a variety of orthopedic conditions who attended our clinic in the study period (*n* = 5352) and (ii) with those patients whose orthopedic conditions did not involve the knee, to exclude any bias related to the knee site (*n* = 1805). These data are reported in Table 4.

## 4. Discussion

The chief finding of this study is the dataset of the demographic, clinical, and radiological characteristics of a substantial cohort of patients with primary BME of the knee. To the best of our knowledge, this is the first study describing the natural history of the disease in patients where all known causes of secondary BME have been excluded.

Half of our patients suffered from a thyroid disorder, with odds ratios of 18.5 and 9.8 compared, respectively, with all the patients with a variety of orthopedic conditions attending our clinic in the study period (*n* = 5352) and with those whose orthopedic conditions did not involve the knee (*n* = 1805). The influence of thyroid physiopathology and vitamin D metabolism on BME onset has been described [22], though not with specific reference to the knee joint.

Analysis of our dataset indicated that most patients with BME were slightly overweight middle-aged female smokers with a sedentary lifestyle [22].

We also found that nearly half of the patients had experienced stressful events such as a family bereavement or dismissal from work in the days preceding the sudden onset of knee pain. BME pain also developed in 12.5% of our cohort during the COVID-19 pandemic. These findings suggest its strong psychological impact as a major source of distress and depression. Indeed, several other conditions related to anxiety and stress, such as fibromyalgia and frozen shoulder, have increased during the pandemic [23,24]. All our patients described intractable day and night pain with both passive and active movements as their chief symptom. The pain was rated as 8.5/10 on an NRS scale, regardless of the extent of the area affected by the edema. The strong pain associated with BME takes a heavy toll on mental and emotional wellbeing [25]. We believe that distress may have been a cause of primary BME of the knee in these patients.

Notably, after treatment and radiological resolution of the edema, most patients (93.8%) developed dysesthesia and hypoesthesia in the skin area overlying the site of the previous edema and a considerable reduction in the ipsilateral patellar reflex. These symptoms suggest a role for the nervous system both as a cause of BME and as a factor in pain onset, as hypothesized by several researchers [3,22,26].

These three symptoms have never been described in the literature and indicate that even after radiological resolution of the edema, the knees did not really heal.

All our patients had complete (type C) bone marrow lesions according to Compagnoni et al. [18]. It may therefore be hypothesized that type C primary lesions are more likely to regress with conservative treatment.

With regards to geographic provenance and month of pain onset, most patients came from the north of Italy and reported that pain had arisen in winter. These data suggest that amount of sun exposure and blood vitamin D concentration may confer some protection from BME [22].

Conservative treatment achieved BME resolution or regression in all patients. Our data indicate that NSAIDs are not useful in managing BME of the knee, as recently hypothesized by some colleagues [27], even though they contrast with other findings describing their value in early treatment stages [3,28].

Besides the small patient sample, the chief limitations of this study are that its design cannot establish cause–effect relationships and that its retrospective nature prevents controlling the variables during follow-up.

Further investigation of larger patient samples is warranted to establish the role of endocrinological, psychological, and neurological disorders in BME pathogenesis.

## Figures and Tables

**Table 1:** Inclusion and exclusion criteria.

Inclusion Criteria	Exclusion Criteria
Age 18–75 years	Osteochondritis dissecans
Diagnosis of BME ^1^ of the knee on MRI	Avascular necrosis
Field strength ≥ 3 Tesla	Spontaneous osteonecrosis before or during follow-up
Follow-up > 12 months	Tumor
	Edema induced by trauma or high mechanical stress
	History of knee surgery
	Infection
	Osteoarthritis (Kellgren–Lawrence grade 2, 3, or 4)
	Chondral defect grade 3 or 4 (Outerbridge classification)
	Symptomatic meniscal tears
	Pregnancy
	Anorexia nervosa
	Complex regional pain syndrome
	BME involving only patella

^1^ BME = bone marrow edema.

**Table 2:** BME ^1^ pain intensity according to the NRS ^2^.

Before Treatment	*n* (%)
1–10 NRS, (median (1st–3rd quartile))	8.5 (8; 10)
Sudden	48 (100)
Day and night pain	45 (93.8)
Burning	9 (20.0)
Throbbing	6 (13.3)
During active and passive movement	42 (87.5)
Diffuse pain	42 (87.5)
Weight-bearing ability	18 (37.5)
Duration, months (median (1st–3rd quartile))	4 (3; 4)
**After Treatment**	
Dysesthesia at edema site on palpation	45 (93.8)
Skin hypoesthesia in area overlying the edema	45 (93.8)
Reduced ipsilateral patellar reflex	45 (93.8)

^1^ BME = bone marrow edema; ^2^ NRS = numerical rating scale.

**Table 3:** NRS ^1^ pain intensity before and after treatment of BME ^2^ of the knee.

Median (1st–3rd Quartile)	Before Treatment	After Treatment	Difference
NSAIDs ^3^ group	8 (8; 10)	0 (0; 0)	−8 (−8; −8)
No NSAIDs group	10 (8; 10)	0 (0; 0)	−9 (−10; −7)
Difference between the groups: *p* = 0.464			
Difference between before and after treatment: *p* < 0.001			
Interaction between groups and treatment duration: *p* = 0.106			
*p*-values refer to non-parametric analysis of longitudinal data in factorial experiments			

^1^ NRS = numerical rating scale; ^2^ BME = bone marrow edema; ^3^ NSAIDs = non-steroidal anti-inflammatory drugs.

**Table 4:** Association of BME ^1^ and thyroid disorders.

		BME		
		Yes	No	Odds Ratio (95% CI ^2^)
**Total patients**				
Thyroid disorders	Yes	24	272	18.5 (10.4; 33.0)
	No	24	5032	
**Patients without knee conditions**				
Thyroid disorders	Yes	24	167	9.8 (5.4; 17.7)
	No	24	1638	

^1^ BME = bone marrow edema; ^2^ CI = confidence interval.

## Data Availability

The datasets generated and analysed during the current study are available from the corresponding authors on reasonable request.
